# Tracheal development after left pulmonary artery reimplantation: an individual study

**DOI:** 10.1038/s41598-020-74890-4

**Published:** 2020-10-19

**Authors:** Xiaoyang Hong, Ruijie Li, Zhe Zhao, Jiangheng Guan, Hui Wang, Gang Wang, Xiaohong Liu, Qin Yu, Jun Li, Gengxu Zhou, Zhichun Feng

**Affiliations:** 1 Department of Pediatric Cardiology, BaYi Children’s Hospital, The Seventh Medical Center of PLA General Hospital, The Second Clinical School of Southern Medical University, Beijing, China; 2 School of Physical Science and Technology, Guangxi University, Nanning, China; 3 Department of Neurosurgery, The General Hospital of Central Theater Command of PLA, Wuhan, China; 4 Pediatric Intensive Care Unit, Qilu Children’s Hospital of Shandong University, Jinan, China

**Keywords:** Paediatric research, Respiratory distress syndrome

## Abstract

Pulmonary artery sling (PA sling) often presents as a life-threatening condition requiring urgent surgical correction. We reported 32 cases of PA sling in children who were followed up postoperatively in the past 6 years. All patients with PA slings who were admitted to the hospital from January 2012 to December 2017 and underwent surgery were retrospectively analyzed. The mean age of the 32 patients at repair was 16.97 months (range, 15 days to 128 months). Six patients required ventilator assistance for respiratory failure. All children underwent left pulmonary artery (LPA) reimplantation (n = 32), and 3 patients needed reimplantation slide tracheoplasty (n = 3) due to ventilation weaning failure. Four patients died, 27 patients survived until discharge, and 18 patients were followed up. Pulmonary computed tomography imaging and echocardiography were performed in 18 patients who were followed up. After LPA reimplantation, the tracheal carina area was significantly enlarged compared to that preoperation (*p* = 0.0002). In this follow-up cohort study, 75% of the patients who underwent LPA reimplantation survived until discharge. The survivors had subsequently well-developed pulmonary arteries and tracheas.

## Introduction

Left pulmonary sling (PA sling) was first reported in 1897, and it is still a rare congenital cardiovascular abnormality^1,2^. It is characterized by a left pulmonary artery (LPA) from the right pulmonary artery (RPA) and flow between the trachea and esophagus to the left lung hilum; ~ 1/2–2/3 of PA sling patients were found to have complete tracheal rings, also known as "sling complexes"^1,2^. Patients with PA sling may experience early-onset dyspnea or difficulty swallowing due to abnormal LPA compression of the tracheobronchus and esophagus. However, many patients have “near-death” spells due to congenital bronchial abnormalities. To date, a few reports on PA sling have focused on the choice of surgical approach^3,4^, but there have been no reports of patient development, such as the development of the trachea, height and weight after surgery.

Current viable surgical options include LPA reimplantation and LPA reimplantation + tracheoplasty. Most researchers recommend correcting vascular malformations and tracheal stenosis at the same time under cardiopulmonary bypass through median sternotomy^5–8^. The advantage of the method of correcting cardiovascular malformations and tracheal stenosis is that the problem can be solved with only one surgery. However, the obvious shortcomings of this method are below. First, the postoperative mortality rate of children under one year old is high^9^ for requiring longer cardiopulmonary bypass (CPB) times. Second, there are many early and late complications associated with tracheal anastomosis. Even the most popular slide tracheoplasty approach cannot avoid those complications such as anastomotic leakage, late tracheal softening and tracheal restenosis after tracheoplasty^9–11^. In recent years, some experts have adopted the strategy of single left pulmonary artery transplantation, thereby relieving pressure on the trachea, and obtaining space for the growth and development of the stenosed trachea. Kwak et al. performed left pulmonary artery transplantation alone in 14 PA sling children with tracheal stenosis^12^. However, the authors did not perform a long-term follow-up of tracheal development in these children. For PA sling children with tracheal stenosis, it is still unclear whether left pulmonary artery transplantation alone can promote further growth and development of the stenosed trachea. Cardiovascular computed tomography angiography (CCTA) is considered to contribute to the assessment of congenital heart disease (CHD)^13^, especially when planning for cardiovascular diseases and tracheobronchial abnormalities, so we performed pulmonary artery + 3D bronchial reconstruction evaluations for the followed-up patients.

Here, we report our experience with 32 patients with PA sling who underwent left pulmonary artery reimplantation from 2012 to 2017. According to the preoperation and postoperation tracheal CT, the tracheal area was measured before and after surgery to assess tracheal development. In the end, a comparison of various developmental indicators among children of the same age was carried out.

## Results

### Working flow and basic information of LPA sling

**Figure 1:**
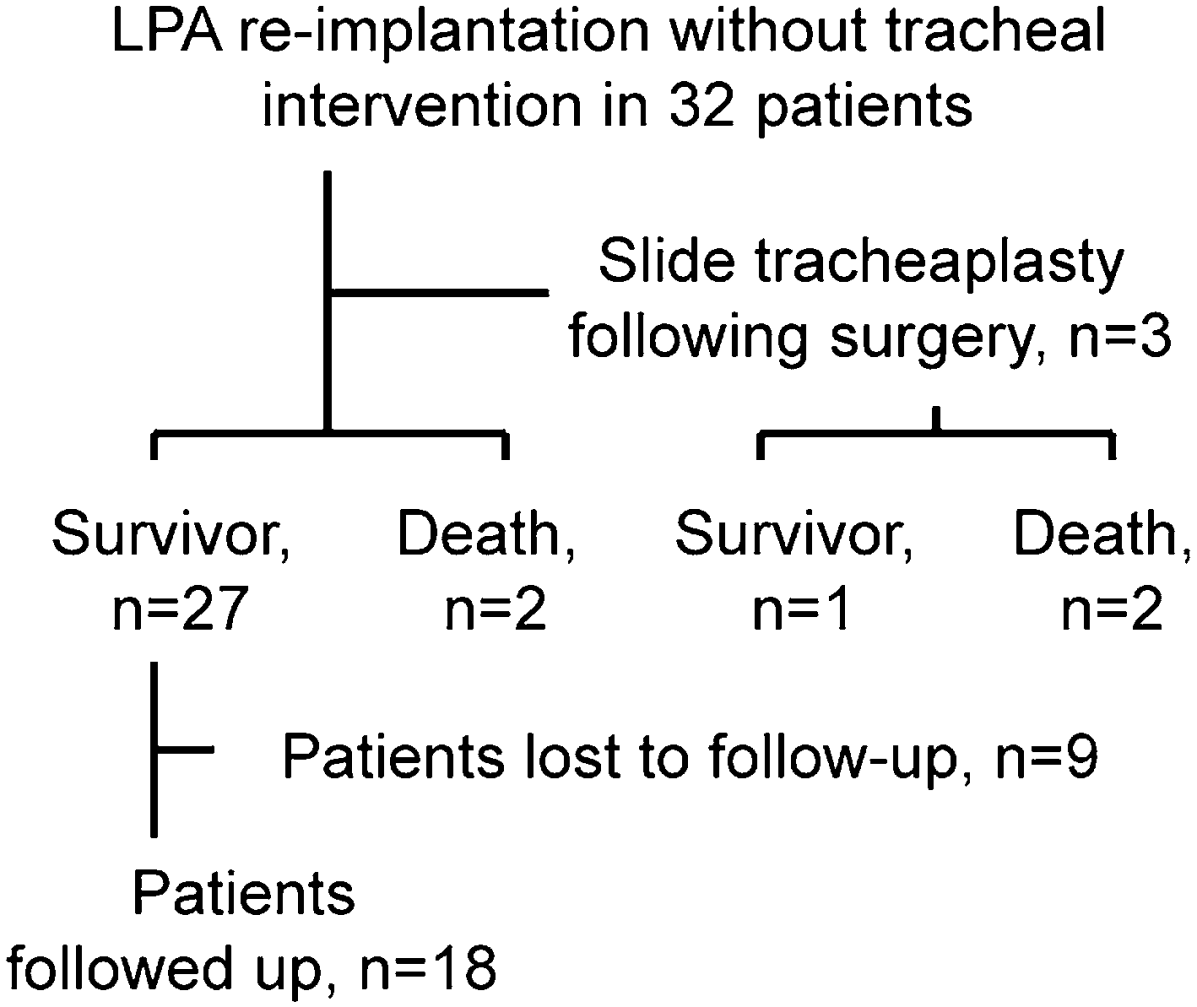
Workflow process to obtain 32 patients with PA sling.

A total of 32 left pulmonary slings diagnosed by echocardiography and CT were retrospectively analyzed from January 2012 to December 2017 (Fig. 1). Thirty-two patients underwent left pulmonary artery reimplantation, and 3 of them underwent slide operations due to ventilation weaning failure. Of the remaining 29 patients, there were 2 deaths, 9 patients were lost to follow up, and 18 patients were successfully followed up.

Among the 18 follow-up patients, 9 were female, and 9 were male (Table 1). The average age at the time of surgery was 16.97 months (0.5–128 months), and the body weight was 8.62 kg (3.6–25 kg). Six patients also had other congenital heart disease, and other congenital heart disease repairs were performed simultaneously during left lung artery transplantation. Based on the symptom discrimination standard of previous study^14^, there were 15 patients with moderate symptoms such as pneumonia and hypoxemia and 3 patients with severe symptoms who needed mechanical ventilation. The duration of follow-up for all patients was 25.23 months (3–65 months).

**Table 1:** Basic information on 32 patients with PA Sling.

Clinic information	Total (%)
No. of patients	18 (100%)
**Sex**	
Male	9 (50%)
Female	9 (50%)
Age at OR (median, range)	16.97 (0.5–128 mo)
Body weight at OR (median, range)	8.62 (3.6–25 kg)
Associate with CHD	6 (33.33%)
**Symptoms and signs**	
Mild	0 (0%)
Moderate	15 (94.74%)
Severe	3 (5.26%)
Pre-surgery ventilation	3 (21.05%)
Survivors follow-up time	25.23 (3–65 mo)

### Tracheal development

**Figure 2:**
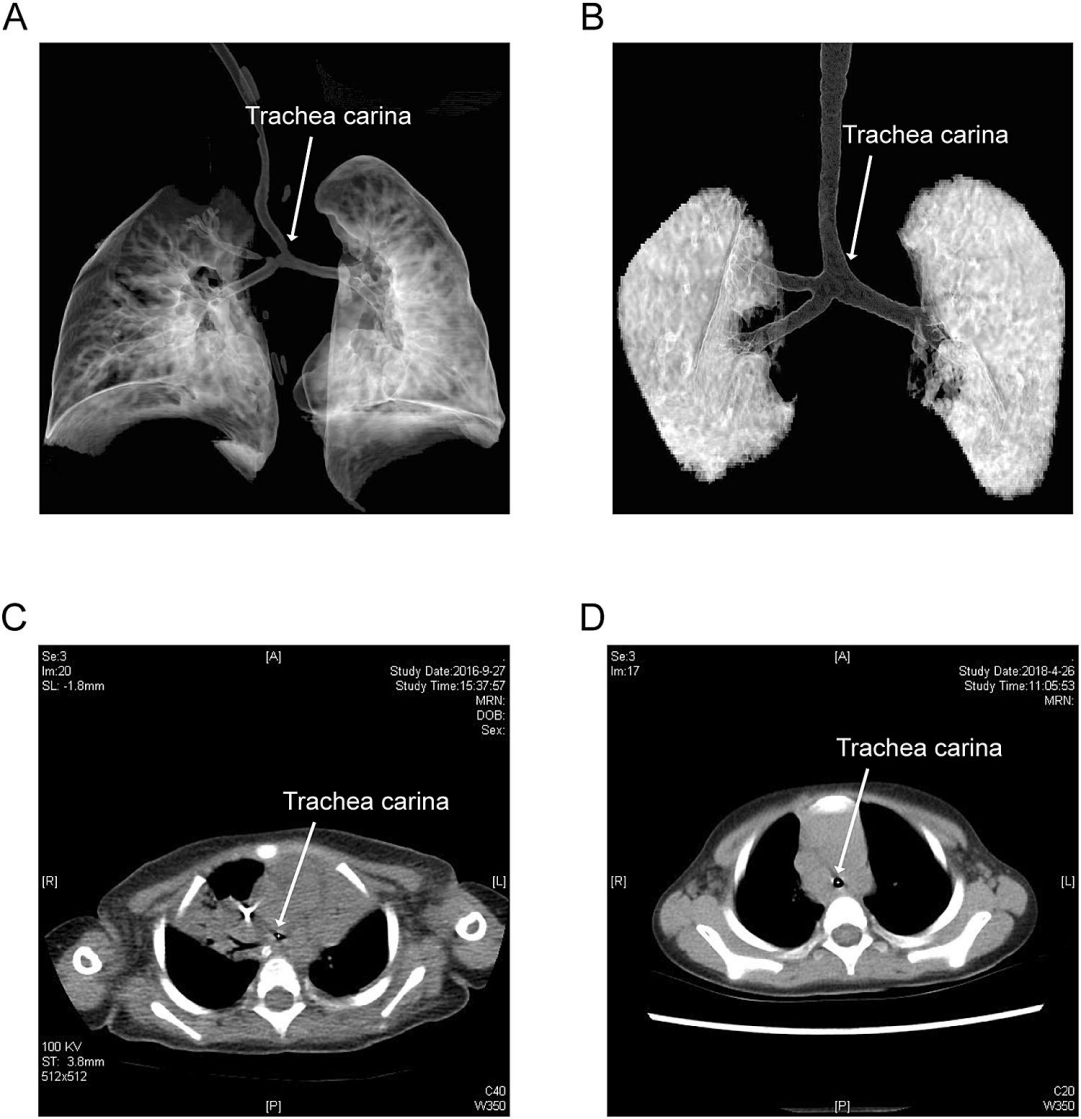
Comparison of tracheal development in an 11-month boy with PA sling. (**A**) Reconstructed 3D image of the trachea and lung before surgery. (**B**) Reconstructed 3D image of the trachea and lung 19 months after surgery. (**C**) Axial image slice of the tracheal carina area (cross in yellow) level before surgery, corresponding to panel A. (**D**) Axial image slice of the tracheal carina area (cross in yellow) 19 months after surgery, corresponding to panel A.

**Figure 3:**
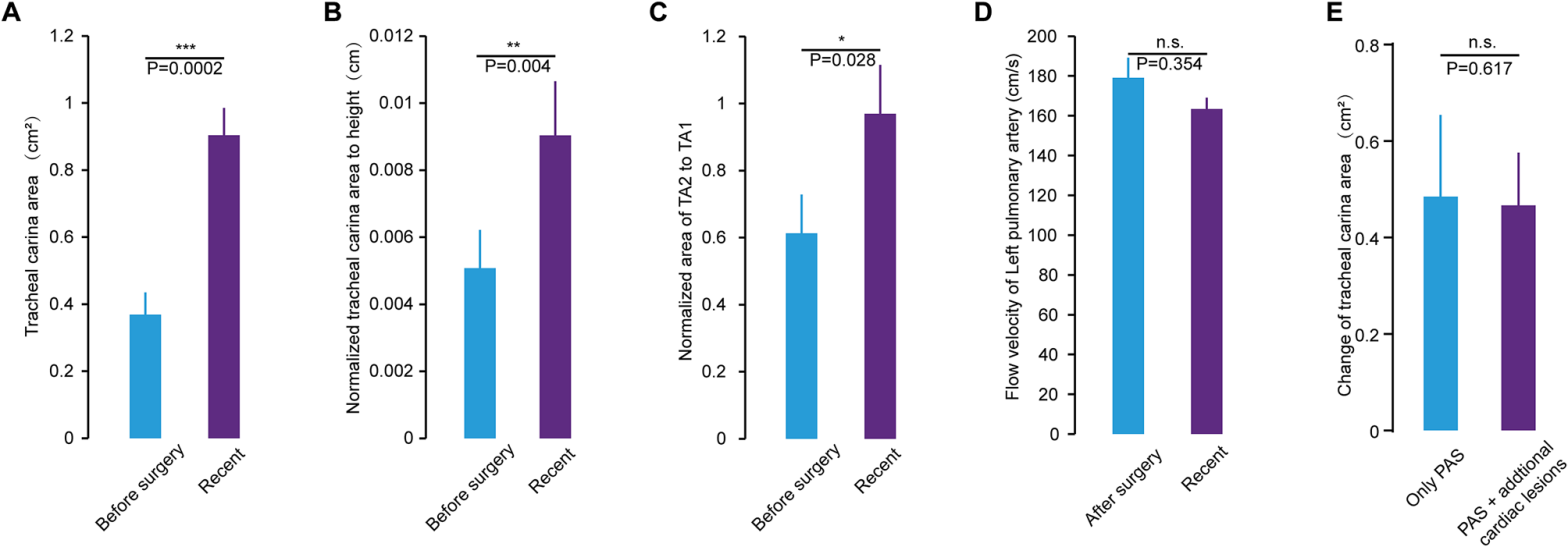
Statistical charts of the tracheal carina area and left pulmonary artery blood flow velocity. (**A**) Statistics show a significant increase (***p = 0.0002) in the tracheal carina area between the preoperative and recent values. (**B**) Statistics show a significant increase (**p = 0.004) in the normalized tracheal carina area between the preoperative and recent values. The tracheal carina area data are normalized to height. (**C**) Statistics show differences (*p = 0.028) in the normalized tracheal carina index between the preoperative and recent values. The tracheal carina area (TA2) data are normalized to the tracheal area of the thoracic entrance (TA1). (**D**) There were no significant differences (*p* = 0.354) in the flow velocity of the LPA between the postoperative and recent periods. (**E**) Statistics show no differences (*p* = 0.354) in the change of tracheal carina area between the patients with only PAS (n = 12) and PAS + additional cardiac lesions (n = 6).

Due to the abnormal anatomical compression of the left pulmonary sling, the development of the trachea underneath is affected, and the narrow tracheal area is the key limitation for air circulation dynamics in the trachea. According to our cases, most of the tracheal compression sites were concentrated above the tracheal carina, so we chose to measure tracheal development with CT scans of the cross-sectional area of ​​the tracheal carina before and after surgery. In Fig. 2, panel A and panel B show the 3D reconstruction of the preoperative and current trachea, respectively, and the white arrows indicate the narrowest part of the trachea (the tracheal carina). Panels C and D show cross-sectional views of the preoperative and current trachea at the narrowest point, respectively. It can be seen that the tracheal carina area increased significantly after surgery, and the tracheal development tended to be normal. According to the statistical analysis, the ​​tracheal carina area increased from 0.3685 to 0.8536 cm^2^ after surgery, which was a significant difference (*p* = 0.0002) (Fig. 3A). To reduce the effects of tracheal development rates at different ages, we used two normalized values, ​​tracheal carina area/height and tracheal carina area (TA2)/tracheal area of the thoracic entrance (TA1), for further comparisons. Among them, the normalized height value increased from 0.0051 cm to 0.0091 cm, which was a significant difference (*p* = 0.004) (Fig. 3B). The normalized value of the tracheal area of the thoracic entrance increased from 0.6151 to 0.9665, which was a statistically significant difference (*p* = 0.028) (Fig. 3C). Moreover, we also tracked the left pulmonary artery blood flow velocity both after surgery and currently, and the flow rate decreased from 178.7692 cm/s to 157.9231 cm/s; although there is no significant difference, there is a downward trend, which indicates that the overly fast blood flow velocity due to the left pulmonary artery was basically resolved (Fig. 3D). In addition, there were no significance differences (*p* = 0.354) in the change of tracheal carina area between the patients with only PAS (n = 12) and PAS + additional cardiac lesions (n = 6) (Fig. 3E).

### Development of weight and height

**Figure 4:**
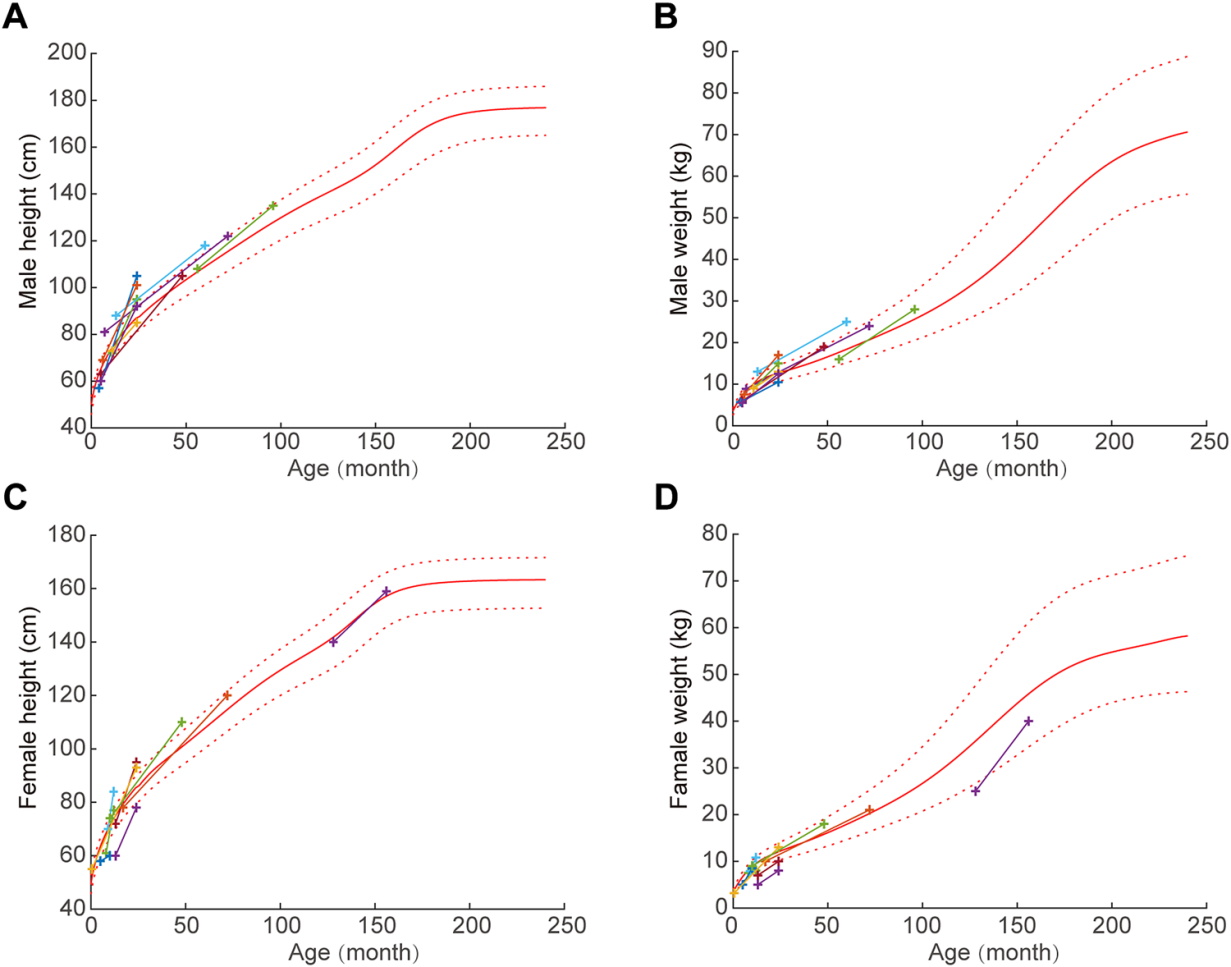
Development curves of the patients. (**A**) Comparison of the preoperative and recent height of male patients. The red curve is the age-height curve of normal male patients, and the dotted lines are 40% above and 40% below the normal value. (**B**) Comparison of the preoperative and recent weight of male patients. The red curve is the age-height curve of normal male patients, and the dotted lines are 40% above and 40% below the normal value. (**C**) Comparison of the preoperative and recent height of female patients. The red curve is the age-height curve of normal female patients, and the dotted lines are 40% above and 40% the normal value. (**D**) Comparison of the preoperative and recent weight of female patients. The red curve is the age-height curve of normal female patients, and the dotted lines are 40% above and 40% the normal value.

Long-term hypoxia and dyspnea caused by tracheal stenosis during early childhood can lead to limited development in PA sling children. We compared the patient's preoperative and current body height and weight. We mainly observed the body weight and height of normal children before and after surgery (the red line is the average of the normal population, and the dotted line is the 10–90% interval of the population) (Fig. 4). Thus, the trend graph of the height of male patients is presented in panel A, and the trend graph of weight gain is presented in panel B. Similarly, the height and weight gain trends of female patients are presented in panels C or D. It can be seen that almost all patients had the same body weight and weight as the normal population, and some even exceeded the development rate of the normal population.

### Long-term survivors

The mean follow-up time for 18 survivors was 25.23 months (age from 16.97 (0.5–128 months) to 43.22 (10–156 months). All patients (18/18, 100%) were asymptomatic at the last follow-up. The survival rate after simple left pulmonary artery reimplantation was 93.10%.

## Discussion

PA sling is a rare, congenital cardiovascular disease in children and is always associated with tracheal stenosis and a complete tracheal ring^15^. Simultaneous LPA reimplantation and tracheoplasty are recommended under CPB in PA sling children with complete tracheal rings and tracheal stenosis^5,8,16,17^. Complete tracheal rings always prevent tracheal development. Tracheoplasty can treat tracheal stenosis directly and ensure ventilation postoperatively. Although the outcome of PA sling has improved in recent decades after the application of slide tracheoplasty, the complications of tracheoplasty still remain a great challenge and include anastomosis leakage, granulation tissue formation and tracheobronchomalacia^17–20^. If tracheobronchomalacia was found postoperatively, then these children had worse outcomes and needed more ventilation time^11^. If granulation of the tracheal tissue emerged postoperatively, then bronchoscopic intervention and a longer ICU stay would be needed^17^. Oshima et al. examined the postoperative complications^11^ of 31 children who underwent PA sling repair; 5 children developed granulation tissue, 3 children had tracheomalacia, and 2 children had anastomotic leakage. To avoid the complications of tracheoplasty, could complete tracheal rings continue grow only by removing PA sling compression? We have found that some PA sling children with complete tracheal rings who only underwent LPA reimplantation achieved good outcomes^4^, but these children need to be further followed up.

In our study, 32 pediatric LPA sling patients with complete tracheal rings and stenosis underwent LPA reimplantation only. A total of 3 children needed another slide tracheoplasty due to extubation failure. Among the other 29 children, 27 survived until discharge, 18 children were followed up, and 9 children were lost to follow-up for social reasons. The total in-hospital mortality rate in this study was 12.5% (Fig. 1). The operative mortality rate in our study was similar to that reported by Yong^21^. In their study, 9 patients underwent LPA reimplantation only and survived until discharge. These 9 patients were followed up for 8 years (3.1–12.7 years) and were asymptomatic. However, there was no evidence to demonstrate that the stenosis site of the trachea can continue to grow with only LPA reimplantation.

It was believed that as long as complete tracheal rings were observed in PA sling children, tracheoplasty must be performed or tracheal developmental would stop. In contrast, in all 18 patients who were followed-up in our study, the tracheal stenosis site was observed to be growing after LPA reimplantation. The statistics show significant increases (*p* = 0.0002) in the tracheal carina area postoperatively without tracheoplasty. To reduce the effects of tracheal development rates at different ages, normalized values ​​of tracheal carina area/height were applied and significantly increased after LPA reimplantation, which means that the tracheal carina area increased more rapidly than body height after LPA reimplantation. The normalized TA1/TA2 value also significantly increased from 0.6151 to 0.9665. The stenosis site of the trachea could grow faster than the normal site in PA sling children after LPA reimplantation. The normalized values ​​of tracheal carina area/height and TA2/TA2 both significantly increased after LPA reimplantation in our study. In conclusion, the stenosis site of the trachea, even in PA sling children with complete tracheal rings, showed accelerated development after LPA reimplantation.

Left pulmonary artery stenosis after reimplantation is known to be a rare complication. In Goldstein and colleagues’ study^22^, they found a high incidence of LPA stenosis after LPA reimplantation in PA sling children. They reported that 45% of their patients needed another intervention for PA stenosis. In contrast, none of children in our study had left PA stenosis according to echocardiographic studies. Similarly, two other studies found a low incidence of severe LPA stenosis after LPA reimplantation^12,22^. There was no significant difference (*p* = 0.354) in the flow velocity of the LPA before and after LPA reimplantation in our study. Although there was no significant difference, our result showed there was a downward trend in LPA blood flow velocity after surgery. This means that the further development of the diameter of LPA may have undergone after reimplantation.

It can be seen that all patients had a normal body height and weight postoperatively compared to the normal population of the same age. Some children even exceeded the development rate of the normal population postoperatively. Long-term hypoxia and dyspnea caused by tracheal stenosis during early childhood can lead to limited body development in PA sling children, and these factors can be significantly improved by accelerated tracheal development after LPA reimplantation. This may explain the accelerated development in body height and weight.

Our experience with LPA reimplantation in 32 PA sling children with tracheal stenosis provides strong evidence in favor of the procedure. The follow-up of 18 children showed promising results regarding growth of the stenosis site in the trachea, even in patients with complete tracheal rings. We recommend that the choice of LPA reimplantation should be made carefully and by considering the patient’s clinical status. All the surgeries in this study were performed by the same surgeon; thus, we were able to eliminate the effects of interoperator variability on the outcome of surgery. This adds further value to our results. However, further investigations are be necessary to confirm which kinds of PA sling patients can truly benefit from LPA reimplantation.

## Materials and methods

### Patient selection and clinical information

This study was approved by the Institutional Review Board (Research Ethics Committee of People's Liberation Army General Hospital Seventh Clinical Center, number: 2018–13). All the informed consents have been obtained from the patients’ parents due to all the patients under 18-year-old. All the methods were in accordance with the relevant guidelines and regulations^23^.

We conducted a retrospective analysis of patients diagnosed with PA sling from 2012 to 2017 from our departmental database. Patients with LPA reimplantation were recruited for further study. The inclusion criteria were: ① pediatric patients under 18 years old; ② with a clear diagnosis of pulmonary sling and tracheal narrowing. The exclusion criteria were: ① the tracheal and pulmonary artery surgery at the first stage; ② patients with chromosomal abnormalities; ③ the patients received CPR during in the hospital. All methods required informed consent in accordance with the relevant guidelines and regulations. We recorded and summarized the clinical information, including sex, time of surgery, and weight at the time of surgery, symptoms, preoperative ventilation, surgical treatment, and prognosis, of patients with PA sling.

### Surgery summary and decision making

The 4th intercostal space was incised and the chest was open. After the LPA freed, it was ligated with silk thread and the distal end of the LPA was blocked with blocking forceps. The LPA was cut 2 mm from the ligation line, and the broken end of the LPA was sutured. After the main pulmonary artery dissociated, the LPA and the main pulmonary artery was made an end-to-side anastomosis. If all the procedure completed, no bleeding was detected and the chest was closed. According to reference^1^, when the diameter of the tracheal is less than 3 mm or the length of narrow section beyond 1/2 of the whole length of the tracheal, the patients needed to receive first stage surgery of both tracheal and pulmonary artery.

### Imaging method and postprocessing method

Due to the 7-year study period, the patients underwent two different CCTA scans using a variety of different CT scanners. Echocardiographic images were reviewed by cardiologists, while CCTA images were reviewed in consensus by two radiologists in our group. CT image postprocessing used artificial image recognition based on custom software on MATLAB to determine the tracheal area at the narrowest section of the trachea.

### Statistical analysis

Descriptive statistics are used to describe the frequency (percentage) of each anomaly. A paired t-test was used to compare the differences between the observations before and after surgery in patients with PA sling. A *p* value < 0.05 was considered to indicate a significant difference. Statistical analysis was performed using MATLAB software (2017, NIH, USA).
